# The Prevalence of Several Risky Driving Behaviors and Associated Crash Risk in Adolescent: A Population-Based Study of Tuscany Region

**DOI:** 10.3389/ijph.2022.1604582

**Published:** 2022-05-27

**Authors:** Vieri Lastrucci, Francesco Innocenti, Chiara Lorini, Alice Berti, Caterina Silvestri, Marco Lazzeretti, Fabio Voller, Guglielmo Bonaccorsi

**Affiliations:** ^1^ Epidemiology Unit, Meyer Children’s University Hospital, Firenze, Italy; ^2^ Epidemiologic Observatory, Regional Health Agency of Tuscany, Firenze, Italy; ^3^ Department of Health Sciences, University of Florence, Firenze, Italy

**Keywords:** public health, Italy, adolescent, risky driving behaviors, road traffic accidents, prevalence study, population-based sample, representative sample

## Abstract

**Objectives:** To evaluate the prevalence of numerous risky driving behaviors (RDBs) and the associated risk of road traffic accidents (RTA) in a population-based sample of adolescent drivers (14–19 years) of Tuscany, Italy.

**Methods:** The frequency of participation -by age and sex- often RDBs were investigated: Multivariable analyses were performed to evaluate the association between RDBs and the risk of RTA and severe RTA.

**Results:** 2,737 adolescents were included in the study. Talking to passenger(s), listening to loud music, speeding, and texting showed the highest weekly participation rates. For all the considered RDBs, the frequency of participation significantly increased with age. Males reported a significantly higher participation in speeding, DUI of alcohol or drugs; while females reported listening to loud music and talking to passenger(s) more frequently. All the considered RDBs were significantly associated with the risk of RTA and severe RTA.

**Conclusion:** The prevalence of RDBs and the associated risk of RTA largely varied in adolescents. Findings provide evidence for tailoring prevention interventions and suggest the need to include common- but traditionally overlooked- RDBs in road safety campaigns.

## Introduction

Road traffic accidents (RTAs) are one of the leading causes of death, disabilities and serious injuries among adolescents [1–3]. Adolescents are the population group that present one of the highest risks of RTA; besides their inexperience with driving tasks, the participation in risky driving behaviors (RDBs) can be identified as one of the main determinants of the high risk of RTA in adolescents [4–9]. Indeed, adolescent drivers seem to participate more frequently than older drivers in a wide variety of RDBs, such as texting or talking to the phone while driving and speeding [10, 11]. Furthermore, the risk of RTA related to the participation in certain RDBs—such as impaired driving states and carrying other passengers–seems to be higher in adolescent drivers than in more experienced drivers [12, 13]. As a result, adolescent drivers are distinctively over-represented in RTA statistics, especially in RTAs related to RDBs such as driving under the influence of alcohol or drugs, distracted driving, and speeding [14–16].

The characterization of RDBs participation in adolescents is essential to orient public health policies and to tailor specific prevention interventions. In this regard, it should be highlighted that–given the differences across countries concerning regulations on adolescent driving and other influencing socio-cultural conditions–research aimed to identify, characterize and–eventually–prevent RDBs in adolescent drivers requires triangulation of evidences from different licensing systems to derive generalizable conclusions [17]. Furthermore, the driving behaviors of male and female adolescent drivers need to be regularly reexamined as changing gender roles may influence the participation in RDBs [18]. However, to date, prevalence studies assessing multiple risky driving behaviors among adolescents on large-scale representative samples remain very limited, especially outside the North American contexts [17, 19–22].

Lastly, as for the role played by the different RDBs in causing RTA in adolescents, some RDBs are extensively examined in the literature, such as speeding, driving under the influence (DUI) of alcohol or illegal drugs, texting and talking on the phone while driving. For these RDBs there is overwhelming evidence of a significant increase of RTA risk [6, 21, 23–26]. On the other hand, other common RDBs - such as eating/drinking, smoking, listening to loud music, talking to passenger(s)- appear to be hardly studied in the literature, and their role in increasing the risk of RTA has still to be elucidated [6, 13, 23, 27].

The present study was carried out in a representative sample of adolescent drivers of Tuscany Region, Italy with the aim of providing Regional estimates of the prevalence of numerous RDBs. Furthermore, the study aimed to evaluate the risk of RTA associated with each RDB. The study findings will help to fill the gap of the paucity of prevalence data on RDBs in adolescent population from the European context. Furthermore, among the examined RDBs, the present study provides the prevalence and the associated crash risks of various RDBs that are scarcely studied in the literature.

This study is based on data from the 2018 EDIT (Epidemiologia dei Determinanti dell’Infortunistica Stradale Toscana—Epidemiology of the determinants of traffic accidents in Tuscany Region) surveillance system and follows and complements a previous research which was aimed to identify profiles of RDBs in adolescents [28].

## Methods

The EDIT surveillance system was approved for research purposes by the Decree of the President of the Council of Ministers of Italy (Decreto del Presidente del Consiglio dei Ministri—DPCM) of 3 March 2017. The study was conducted according to the principles described in the Declaration of Helsinki.

### Study Population

The EDIT surveillance system is aimed to evaluate RDBs and RTAs in a representative sample of adolescents attending the upper secondary schools of the Tuscany Region, Italy. The EDIT surveillance system adopts a repeated cross-sectional survey design (repeated every 3 years). The present study analyzes the data from the 2018 survey (carried out from February to May). Further details concerning the EDIT survey methodology has been described elsewhere [28].

A total of 6,824 students participated (response rate 96.6%) in the 2018 survey, representing 3.55% of the population aged 14–19 in Tuscany Region. For the purpose of the present study only participants who reported to drive at least once a week were considered (2,737). In particular, drivers with a full driving license for the following type of vehicles were considered for the study: moped with an engine capacity below 50cc (minimum driving age 14 years); motorbike of 50cc–125cc (minimum driving age 14 years); motorbike over 125cc (minimum driving age 16 years), and passenger car (minimum driving age 18 years).

### Data Collection and Measurements

Data were collected through a questionnaire administered *via* tablet devices allowing a real-time data collection. In particular, students were asked to fill an anonymous self-administered questionnaire during the school time.

The questionnaire was comprised of 82 questions and had an average completion time of 45 min.

As far as RDBs are concerned, the following RDBs were investigated: driving under the influence (DUI) of alcohol; DUI of recreational drugs; talking on the phone while driving; texting while driving; talking to passenger(s); smoking while driving; eating while driving; listening to loud music while driving; fatigued driving; and speeding. In particular, participants were asked to report the average frequency of participation in RDBs during the course of the previous 12 months with the following response options (except for DUI of alcohol and drugs): never; a few times a month; several times a week; once a day; more than once a day. DUI of alcohol and DUI of drugs were assessed with the following response options: never, once a month, a few times a month, a few times a week, several times a week. Furthermore, the type of motor vehicle used and the average frequency of driving (6–7 times a week, 2–5 times a week, once a week) were evaluated. Lastly, RTA was investigated with the following question “have you ever had a RTA while driving a vehicle in life (excluding minor crashes with very limited material damage)?”. Furthermore, participants were asked to report the number of severe RTA (i.e., RTA that caused the hospitalization of the driver) occurred while they were driving a vehicle in life.

### Statistical Analysis

Data were weighted (age, sex and sub-regional administrative areas) to more closely align the sample with official figures for population aged 14–19 of Tuscany Region.

Demographic variables included age and sex. For each RDB, the frequency of participation was grouped in the following three categories: never, few times a month, several times a week. Frequency of participation was reported as percentage and 95% CI. Associations between the frequency of participation in RDBs and demographic characteristics (age and sex) were investigated by chi-square’s test and ANOVA for weighted data. Multivariable logistic regression models were performed to evaluate the association between RDBs and the risk of RTAs. In particular, one model considered the RTA occurred while driving a vehicle in life (no RTA in life vs. have ever had a RTA in life) as dependent variable. A second model considered the risk of a severe RTA (i.e., no severe RTA in life vs. one or more severe RTAs in life) as dependent variable; although the severe RTA variable was collected as count data, this variable was dichotomized because the number of students reporting two or more severe RTAs was very low. All the models were adjusted by age, sex, average driving frequency and type of motor vehicle used. For each analysis, an α level of 0.05 was considered as significant.

The software used for the sample size calculation was Epi Info, and STATA (Version 15.0; Stata Corporation) was used for data analyses.

## Results

The study sample consists of a total of 2,737 students who reported to drive at least once a week (40.5% of the EDIT survey participants). Unweighted and weighted data of the sample characteristics are reported in Table 1. The mean age of the sample was 16.50 ± 1.39 years and males represented 68.2% of the sample. As for the average driving frequency, 64.6% of the sample reported to drive 6 days a week/every day, whereas only 9.3% drove 1 day a week. A total of 936 participants (33.2%) reported to have had at least one RTA while driving, during the course of their life.

**TABLE 1:** Demographic characteristics and driving behaviors of the study population (N = 2.737) (Epidemiologia dei Determinanti dell’Infortunistica Stradale Toscana Study, Tuscany Region, Italy, 2018).

	N	Weighted			
		N weighted	% (95% CI^a^)	Proportion of male (%)	Mean age (95% CI^a^)
Sex^b^					
Male	1,917	37,840	68.2 (67.8–68.6)		16.42 (16.41–16.43)
Female	820	17,641	31.8 (31.4–32.2)		16.67 (16.66–16.68)
Avarege driving frequency** ^c^					
6 day a week/every day	1,799	35,853	64.6 (64.2–65.0)	72.6	16.59 (16.58–16.60)
2–5 days a week	700	14,456	26.1 (25.7–26.4)	60.8	16.38 (16.37–16.39)
1 day a week	238	5,172	9.3 (9.1–9.6)	58.9	16.21 (16.20–16.22)
Talking on the phone while driving^c^					
Never	1,962	41,206	77.5 (77.2–77.8)	67.4	16.33 (16.32–16.35)
Few times a month	348	6,387	12.0 (11.7–12.3)	71.2	16.93 (16.91–16.94)
Several times a week	310	5,576	10.5 (10.2–10.7)	67.0	17.25 (17.24–17.26)
Texting while driving^c^					
Never	1,644	35,059	66.7 (66.3–67.1)	67.0	16.23 (16.22–16.24)
Few times a month	469	8,889	16.9 (16.6–17.2)	70.5	16.92 (16.91–16.93)
Several times a week	476	8,594	16.4 (16.0–16.7)	67.5	17.16 (17.15–17.17)
Smoking while driving^c^					
Never	2,129	44,043	83.0 (82.7–83.3)	67.8	16.39 (16.38–16.41)
Few times a month	173	3,527	6.6 (6.4–6.9)	62.6	16.84 (16.83–16.85)
Several times a week	309	5,500	10.4 (10.1–10.6)	67.9	17.21 (17.20–17.22)
Eating while driving^c^					
Never	2,038	42,101	80.1 (79.8–80.5)	67.0	16.42 (16.41–16.43)
Few times a month	318	6,013	11.4 (11.2–11.7)	70.4	16.97 (16.95–16.98)
Several times a week	233	4,415	8.4 (8.2–8.6)	68.6	16.62 (16.61–16.63)
Talking to passenger(s)* ^c^					
Never	753	16,505	31.9 (31.5–32.3)	69.7	15.72 (15.71–15.73)
Few times a month	450	9,677	18.7 (18.4–19.0)	71.6	16.41 (16.40–16.42)
Several times a week	1,356	25,532	49.4 (49.0–49.8)	64.3	17.08 (17.07–17.09)
Listening to loud music while driving* ^c^					
Never	1,390	29,555	56.5 (56.1–56.9)	70.7	16.16 (16.15–16.17)
Few times a month	280	5,759	11.0 (10.7–11.3)	65.7	16.71 (16.69–16.72)
Several times a week	919	17,022	32.5 (32.1–32.9)	62.0	17.06 (17.05–17.07)
Fatigued driving^c^					
Never	1,536	31,953	60.9 (60.5–61.3)	68.0	16.32 (16.31–16.33)
Few times a month	880	17,116	32.6 (32.2–33.0)	68.0	16.84 (16.83–16.85)
Several times a week	175	3,385	6.5 (6.2–6.7)	68.0	16.77 (16.76–16.78)
Speeding* ^c^					
Never	869	18,050	33.6 (33.2–33.9)	68.8	16.36 (16.35–16.38)
Few times a month	969	19,887	37.0 (36.6–37.4)	71.2	16.44 (16.43–16.45)
Several times a week	816	15,863	29.5 (29.1–29.9)	62.8	16.78 (16.77–16.79)
Driving under the influence of alcohol* ^c^					
Never	2,271	46,536	87.4 (87.1–87.7)	66.8	16.45 (16.44–16.46)
Few times a month	275	5,293	9.9 (9.7–10.2)	73.3	16.92 (16.91–16.93)
Several times a week	78	1,417	2.7 (2.5–2.8)	83.5	16.95 (16.94–16.96)
Driving under the influence of drugs* ^c^					
Never	2,346	48,024	88.6 (88.3–88.8)	67.2	16.46 (16.44–16.47)
Few times a month	192	3,770	7.0 (6.7–7.2)	71.1	16.88 (16.87–16.89)
Several times a week	132	2,430	4.5 (4.3–4.7)	82.6	16.99 (16.98–17.0)
Road traffic accidents in life (while driving)* ^c^					
No	1,801	37,045	66.8 (66.4–67.2)	64.6	16.37 (16.36–16.38)
Yes	936	18,436	33.2 (32.8–33.6)	75.4	16.76 (16.75–16.77)
Severe road traffic accidents in life (while driving)^c^					
No	2,404	49,027	88.6 (88.3–88.8)	67.6	16.45 (16.44–16.46)
One or more	327	6,320	11.4 (11.2–11.7)	72.2	16.91 (16.90–16.92)

a CI: confidence interval.

b ANOVA for age, *p* < 0.05.

c ANOVA for age, *p* < 0.001.

*Chi^2^ test for sex, *p* < 0.05; ** Chi^2^ test for sex, *p* < 0.001.

Unweighted and weighted data for all the considered RDBs are reported in Table 1. The highest percentages of weekly participation were observed in the following RDBs: talking to passenger(s) (49.4%, 95%CI 49.0-49-8), listening to loud music while driving (32.5%, 95%CI 32.1–32.9), and speeding (29.5, 95%CI 29.1–29.9) (Table 1). The RDBs that presented the highest percentages of non-participation were DUI of alchol and DUI of drugs; during the last year, 12.6% and 11.5% of the participants reported DUI of alcohol or drugs, respectively; in particular, a several times a week frequency of DUI of alcohol or drugs was observed in 2.7% (95%CI 2.5–2.8) and 4.5% (95%CI 4.3–4.7) of the sample, respectively. talking on the phone and texting showed a moderate participation among participants: 77.5% (95%CI 77.2–77.8) and 66.7% (95%CI 66.3–67.1) of the participants reported a non-participation in these RDBs during the last year, respectively; whereas, 10.5% (95%CI 10.2–10.7) and 16.4% (95%CI 16.0–16.7) of the sample reported talking on the phone and texting several times a week, respectively.

As far as age differences in RDBs participations are concerned, the mean age of participants significantly increases moving from “never” to “a few times a month” frequency of participation and from “a few times a month” to “several times a week” frequency of participation, this trend was observed in all the RDBs, with the exception of eating while driving. As for sex differences in RDBs participation, moving to higher frequencies of participation, a higher proportion of males reported DUI of alcohol or drugs, while a higher proportion of females reported listening to loud music while driving. Significant sex differences were also observed for speeding and talking to passanger(s), but no clear trend was observed in the frequency of participation.

Table 2 reports the results of the logistic regression models for the risk of RTA and severe RTA. As for the risk of RTA, all the examined RDBs resulted to be significantly associated with the risk of RTA. In particular, the odds of RTA increased with the frequency of participation in the RDB, with participants with a several times a week frequency of participation showing the highest odds of RTA in all the considered RDBs. Frequent (i.e., several times a week) engagement in DUI of alcohol was associated with more than triple the odds, and a similar frequency of talking on the phone or speeding was associated with almost triple the odds, of involvement in RTAs. The risk of severe RTA was significantly associated with the participation in all the RDBs, with the odds ratios increasing with the frequency of participations for all the RDBS except for fatigued driving and eating while driving. For all the RDBs the odds ratios of severe RTA were higher than the ones observed for RTA risk, and the RDBs that showed the highest odds ratios of severe RTA at several times a weekly frequency of participation were talking on the phone, DUI of alcohol, and smoking while driving, the odds of involvement in RTA were increased of about four to five times in these RDBs.

**TABLE 2:** Logistic regression models for the risk of road traffic accident (model 1) and severe road traffic accident (model 2), adjusted by sex, age, average frequency of drive, and type of motor vehicle driven (Epidemiologia dei Determinanti dell’Infortunistica Stradale Toscana Study, Tuscany Region, Italy, 2018).

	Model 1			Model 2		
	Odds ratio	95%CI^a^	p	Odds ratio	95%CI^b^	p
Talking on the phone while driving						
Never	1			1		
Few times a month	1.65	1.25–2.19	<0.001	2.20	1.41–3.42	<0.001
Several times a week	2.89	2.10–3.98	<0.001	5.12	3.26–8.05	<0.001
Texting while driving						
Never	1			1		
Few times a month	1.41	1.09–1.83	0.010	1.70	1.14–2.54	0.009
Several times a week	2.22	1.68–2.95	<0.001	3.68	2.44–5.56	<0.001
Smoking while driving						
Never	1			1		
Few times a month	1.78	1.24–2.55	0.002	2.08	1.28–3.37	0.003
Several times a week	2.71	1.99–3.69	<0.001	3.82	2.53–5.74	<0.001
Eating while driving						
Never	1			1		
Few times a month	1.64	1.23–2.18	0.001	2.92	1.99–4.28	<0.001
Several times a week	1.94	1.36–2.77	<0.001	2.66	1.59–4.45	<0.001
Talking to passenger(s)						
Never	1			1		
Few times a month	1.51	1.13–2.01	0.005	1.33	0.83–2.16	0.233
Several times a week	1.85	1.41–2.42	<0.001	2.63	1.73–4.00	<0.001
Listening to loud music while driving						
Never	1			1		
Few times a month	1.83	1.34–2.49	<0.001	1.72	1.08–2.73	0.022
Several times a week	1.97	1.56–2.49	<0.001	2.61	1.85–3.67	<0.001
Fatigued driving						
Never	1			1		
Few times a month	1.96	1.59–2.42	<0.001	2.76	2.03–3.75	<0.001
Several times a week	2.09	1.43–3.04	<0.001	2.31	1.36–3.94	0.002
Speeding						
Never	1			1		
Few times a month	1.80	1.39–2.34	<0.001	2.07	1.33–3.22	0.001
Several times a week	2.77	2.13–3.61	<0.001	3.66	2.37–5.66	<0.001
Driving under the influence of alcohol						
Never	1			1		
Few times a month	2.08	1.52–2.85	<0.001	2.58	1.72–3.86	<0.001
Several times a week	3.43	1.96–5.98	<0.001	4.16	1.87–9.28	<0.001
Driving under the influence of drugs						
Never	1			1		
Few times a month	2.54	1.78–3.62	<0.001	2.78	1.74–4.45	0.000
Several times a week	2.44	1.57–3.79	<0.001	2.85	1.59–5.08	0.000

a CI: confidence interval.

b SD: standard deviation.

## Discussion

The aim of this study was to evaluate the prevalence of ten RDBs in a large representative population-based sample of adolescents of Tuscany Region, Italy. Alongside the frequency of these RDBs, the aim of the study was to analyze their association with the risk of RTA. Results of the study showed that the frequency with which adolescent drivers reported engaging in various unsafe and risky driving practices largely varied according to the RDB considered. For most of the considered RDBs, results showed that a considerable proportion of adolescent drivers reported a high frequency of participation. Furthermore, for all considered RDBs the frequency of participation was positively associated with age, and a significant association with sex was found for DUI of alcohol and drugs, speeding, talking to passenger(s) and listening to loud music while driving. Lastly, for all the considered RDBs, results of the multivariable logistic regression models showed that adolescents with high frequency of participation had a significant increase in the risk of RTA and severe RTA and that this association had a positive trend, with higher frequency of participation corresponding to higher crash risk.

The frequency with which adolescent drivers reported engaging in various unsafe and risky driving practices largely varied according to the RDB considered. As far as talking on the phone and texting are concerned, a relevant proportion of adolescents reported to participate in these RDBs, with around half of these reporting a several times a week frequency of participation. These frequencies of participation result to be lower than those reported by studies carried out in the United States [17, 22] and higher than other regional estimates from Canada [19, 21]. Past research on sex and age differences in talking on the phone and texting among adolescents is inconclusive, with some studies reporting males and older adolescents more frequently engaged and others reporting no differences [17, 19, 21, 22]. Consistent with some of these studies, our results showed that the frequency of talking on the phone and texting was not associated with sex but was positively associated with age. As for impaired driving, the prevalence of DUI of alcohol or drugs resulted to be relatively high, especially among males and older adolescents; these findings are consistent with prevalence studies from other countries [20, 21].

While confronting our data with the literature, it should be underlined that large population-based studies assessing the prevalence of multiple RDBs among adolescents are scarce, and most of them are based on regional or national population samples from the United States or Canada [17, 19]. Furthermore, for some of the RDBs examined by our study–i.e., eating/drinking, smoking, listening to loud music, fatigued driving, talking to passenger(s)–our results may be considered among the first attempts to evaluate their prevalence in a representative sample of adolescent drivers. These RDBs showed a high frequency of participation, with a considerable proportion of adolescents reporting engaging them at least few times a month. A possible explanation for this high frequency is that most of these RDBs can be considered more acceptable from a social and normative point of view and that they are less frequently targeted by prevention interventions and safety campaigns [29].

As for demographics difference in the frequency of participation in RDBs, our results showed a common trend of increasing prevalence over the course of the teenage years for all the RDBs. This trend is consistent with a reasonable body of research suggesting that younger adolescent drivers do not engage in as much risk behavior as slightly older adolescent and young adult drivers [18, 30]. This finding suggests that adolescents should not be considered as a unique target group for prevention interventions and that—given the fact that health-promotion efforts are best aimed when adolescents are ready to receive the message but prior the problematic habit being established [31]–adolescents should be involved in road safety interventions since early phases of adolescence. As for sex differences in the frequency of participation in RDBs, results of our study showed that males engage more frequently in DUI of alcohol or drugs. These findings probably indicate a possible tendency for male adolescents to be involved in reckless behaviors that are more socially and legally proscribed [28, 32]. On the other hand, females reported listening to loud music and talking to passenger(s) more frequently than males, a finding that may suggest a more “normative” risky driver profile associated with female sex [28, 32].

For all the RDBs considered by the study, our results showed that the participation in RDBs was related with an increased risk of RTA/severe RTA and that this risk increases as the frequency of participation increases. While for some of the examined RDBs–i.e., speeding, texting, talking on the phone, impaired and fatigued driving- it is already known in the literature that they do indeed increase crash risk, for other of the examined RDBs—i.e., listening to loud music while driving, talking to passenger(s), eating and smoking - there is scarce and inconsistent evidence on their riskiness. Our findings regarding the association of several RDBs with a significantly increased crash risk—in particular for severe RTA–provide support for policies limiting the participation in these behaviors and for graduated licensing requirements for adolescents.

As far as eating and smoking while driving behaviors are concerned, these RDBs are distracting behaviors that place visual-manual demands on the drivers and take a driver’s eyes away from the forward roadway. Although these two RDBs are overlooked in the literature, our results confirm that it is reasonable to assume that they significantly increase crash risk, as it is identified for other—more explored—visual-manual distracting behaviors [6, 33, 34]. Indeed, in our study participating in eating or smoking while driving implied a crash risk of a similar magnitude of participating in texting or talking on the phone. These findings on common—but traditionally overlooked RDBs—highlight the need to include and target distracting behaviors as a whole in prevention interventions and road safety campaigns.

As for talking to passenger(s) and listening to loud music, these behaviors are primarily cognitive secondary tasks performed while looking at the road, i.e., tasks that do not place apparent visual-manual demands on the drivers. It is interestingly to note that our results showed that these RDBs placed a risk of RTA, although lower than the other RDBs considered. In this regard, risk associated with performance of cognitive secondary tasks are not well understood in adolescents; however, evidence from naturalistic studies have found little or no association with crash risk in adult population [34–36]. It could be argued that cognitive distractions increase the crash risk in adolescent drivers because of their inexperience. This hypothesis is supported by evidence from studies on visual-manual distracting behaviors, in which adolescent drivers present a higher risk of RTA compared with older drivers [6]. The results on an increased crash risk associated with other passengers support the premise that passenger presence and their number should be introduced gradually such as with licensing policies that limit the presence and number of young passengers for young drivers.

This is one of the first studies providing Regional estimates of the prevalence of numerous and varied set of RDBs in a large and representative sample of adolescent drivers to date; this together with the high participation rate of the study, should be considered as the strengths of this study. Nevertheless, the study has several limitations. As far as the assessment of RTA are concerned, it should be pointed out that the classification of severe RTAs considered only those in which the driver was injured, therefore data reported by our study cannot be considered the prevalence of all the possible severe RTA that may have occurred in adolescent drivers. Furthermore, since the study was based on self-reported survey questionnaire, results may have been influenced by a recall and social desirability bias of the participants. However, it has been reported that the role that such biases play is marginal in self-reporting driving behaviors [37, 38]. Furthermore, the survey was self-administered and anonymous, this may have further limited the potential social desirability bias. Lastly, it should be pointed out that the design of the study does not allow to establish temporal links between RDBs and RTA. Therefore, especially for the less explored RDBs, further evidences from naturalistic driving studies are needed to provide precise crash risk estimates.

Our study showed that the prevalence of RDBs and the associated risk of RTA largely varied in adolescents. Findings provide evidence for tailoring prevention interventions and suggest the need to include common- but traditionally overlooked- RDBs in road safety campaigns in order to effectively address this major public health challenge in adolescent population.

### Conclusion

RTA are a major public health issue in adolescents, and the characterization of RDBs participation in adolescents is essential to orient public health policies and to tailor specific prevention interventions. The study found that a considerable proportion of adolescent drivers reported a high frequency of participation in various unsafe and risky driving practices. In particular, the study showed that the prevalence of RDBs largely varied according to the specific RDB, age and sex. Furthermore, the study found that all the considered RDBs are associated with an increased risk of RTA, including those RDBs that are commonly performed—but traditionally overlooked by the literature and road safety campaigns. These findings support the premise that licensing policies should limit the presence of young passengers for adolescent drivers and suggest the importance to broaden road safety campaigns and prevention intervention to also include common- but traditionally overlooked- RDBs.
